# Deletion of miR-15a inhibited glioma development via targeting Smad7 and inhibiting EMT pathway

**DOI:** 10.18632/aging.203684

**Published:** 2021-11-11

**Authors:** Yanfeng Guo, Xiaopeng Gao, Shien An, Xin Li, Lekun Pan, Hongyan Liu, Jixiang Liu, Jianzhou Gao, Zhihuang Zhao, Gang Li, Yonggang Han, Yabin Li, Zhisheng Ji

**Affiliations:** 1 Department of Neurosurgery, The First Hospital of Handan, Handan, Hebei Province, China; 2 Department of E.N.T, The First Hospital of Handan, Handan, Hebei Province, China; 3 Third Department of Neurosurgery, Cangzhou Central Hospital, Cangzhou, Hebei Province, China

**Keywords:** glioma, miR-15a, Smad7, EMT, tumorigenesis

## Abstract

In the present study, we found the expression of miR-15a-5p (miR-15a) was increased in glioma tissues, and we further explore the underlying mechanism of miR-15a in glioma progression. Microarray analysis used to identify the differentially expressed microRNAs (miRNAs) in glioma tissues. The expression of miR-15a in glioma tissues and cell lines was tested by qRT-PCR. Luciferase assay was used to determine the binding between miR-15a and Smad7. Wound healing and transwell assay were used to examine the role of miR-15a/Smad7 in SHG139 cells. Western blot was used to detect the protein level of Smad7 and epithelial-mesenchymal transition (EMT) markers. A tumor formation model in nude mice was established to measure the role of miR-15a *in vivo*. MiR-15a was significantly increased in glioma tissues and cells, which indicated a poor prognosis of glioma patients. MiR-15a mimics induced miR-15a level in SHG139 cells, and promoted the malignancy of SHG139 cells, while miR-15a inhibitor showed the opposite effects. Luciferase assay indicated that Smad7 was the direct target of miR-15a, and Smad7 was down-regulated in glioma tissues. Functional experiments revealed that miR-15a inhibitor inhibited the EMT pathway and the migration and invasion of glioma cells, but the silencing of Smad7 reversed the effects of miR-15a inhibitor in EMT pathway and glioma progression. Finally, we performed animal experiments to verify the role of miR-15a *in vivo*. Present study showed that deletion of miR-15a inhibited the activation of EMT signaling via targeting Smad7, thus suppressed the tumorigenesis and tumor growth of glioma.

## INTRODUCTION

Glioma is the most common primary malignant central nervous system tumor [1]. At present, it is believed that the main pathogenic factors are genetic factors and carcinogenic environmental factors. According to the investigation, the number of glioma patients can increase by about 14000 cases every year, among which the incidence rate of the elderly is relatively high, and the malignant degree of glioblastoma is the highest [2]. For GBM, even if the maximum safe resection was given with adjuvant radiotherapy and chemotherapy, the overall survival time was only about 14 months. About 89% of patients treated with temozolomide combined with radiotherapy died within 5 years [3, 4]. Although great progress has been made in surgical resection, radiotherapy and chemotherapy in recent years, the overall prognosis of gliomas is still poor [5]. At present, the level of medical treatment is still unable to cure glioma completely, and various treatments will destroy part of the normal brain tissue, the survival rate and quality of life of patients have not been significantly improved. Therefore, it is important to identify the relevant therapeutic targets for the study of the pathogenesis of glioma.

Recent studies have found that miRNA plays a crucial role in regulating tumor cell growth and proliferation [6]. With the progress of transcription, miRNA can bind to the complementary sequence of the target gene and inhibit the expression of target genes by inhibiting protein translation or affecting the stability of target gene miRNA [7]. Many studies have revealed the role of miRNA in the mechanism of tumorigenesis [8]. These miRNAs play the role of tumor-promoting factor or tumor suppressor to some extent by regulating the expression of proteins with regulatory function [9]. The high-throughput detection method was used to detect the differential expression profile of miRNA in different tumor tissues, which further revealed the important role of these miRNA in tumors [10]. With the deepening of the research, some important roles of miRNA are revealed. Previous studies have found that miRNA is involved in various biological processes, including development, differentiation, cell proliferation and tumorigenesis. In addition, miRNA is involved in the occurrence and development of many tumors, including prostate cancer, breast cancer, colon cancer and so on [11, 12]. In gliomas, miR-21 can inhibit the expression of PDCD4, thus inhibit the apoptosis of glioma cells and promote cell proliferation and invasion [13]. The expression of miR-132 is decreased in glioma tissues and cells, and is related to the malignant grade of glioma. Overexpression of miR-132 can inhibit the growth of glioma cells [14].

Epithelial-mesenchymal transformation (EMT) signal pathway is one of the important signal pathways to regulate tumor development [15]. After the occurrence of EMT, the adhesion, polarity and cytoskeleton between cells change, along with the enhancement of the migration ability of epithelial cells and the morphological changes to interstitial cells [16]. Therefore, tumor cells are more likely to fall off from the primary site and then transfer to other tissues and organs, resulting in tumor invasion and metastasis. Some studies have pointed out that overexpression of miR-106b in breast cancer can inhibit the expression of Smad7, further enhance TGF-β signal pathway, lead to the occurrence of EMT and promote tumor progression [17]. In addition, EMT also plays an important regulatory role in the occurrence and development of gliomas. USP18 could interact with Twist1, and increased the activity of EMT pathway, which promoted glioma growth [18].

In the present study, we found the expression of miR-15a was increased in glioma tissues, and we further explore the underlying mechanism of miR-15a in glioma progression.

## RESULTS

### miR-15a was up-regulated in glioma tissues

**Figure 1:**
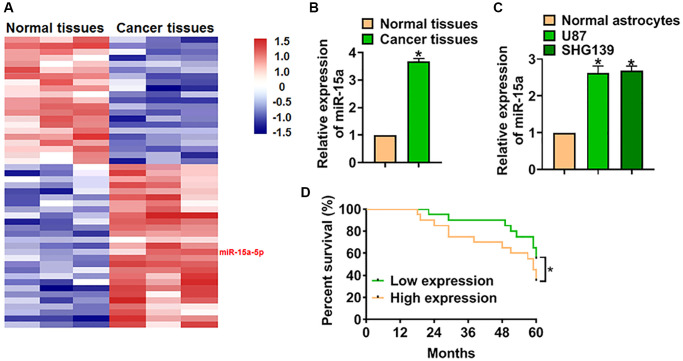
**High expression of miR-15a indicated a poor prognosis of glioma.** (**A**) Microarray analysis used to identify the differential expressed miRNAs in 3 paired glioma tissues. (**B**) The mRNA expression of miR-15a in adjacent normal tissues and cancer tissues of glioma was detected by qRT-PCR. (**C**) MiR-15a level in normal astrocytes and glioma cell lines (U87 and SHG139) was measured. (**D**) The overall survival rate of glioma patients with high or low expression of miR-15a. Data were expressed as mean ± SD.^*^P < 0.05.

We used microarray analysis to identify the differential expressed miRNAs in glioma tissues, and the data showed that miR-15a-5p (miR-15a) was significantly increased in glioma tissues than normal tissues (Figure 1A). PCR analysis also indicated miR-15a was up-regulated in glioma tissues (Figure 1B). Moreover, we detected miR-15a level in normal astrocytes and glioma cell lines (U87 and SHG139), and found miR-15a was more expressed in glioma cell lines (Figure 1C). The expression of miR-15a in U87 and SHG139 cells was no different, and we chose SHG139 cells in the following experiments. Then, we analyzed the overall survival rate of patients with high or low miR-15a expression. The data indicated that patients with low miR-15a expression had a higher survival rate (Figure 1D).

### Deletion of miR-15a inhibited the malignancy of glioma cells

**Figure 2:**
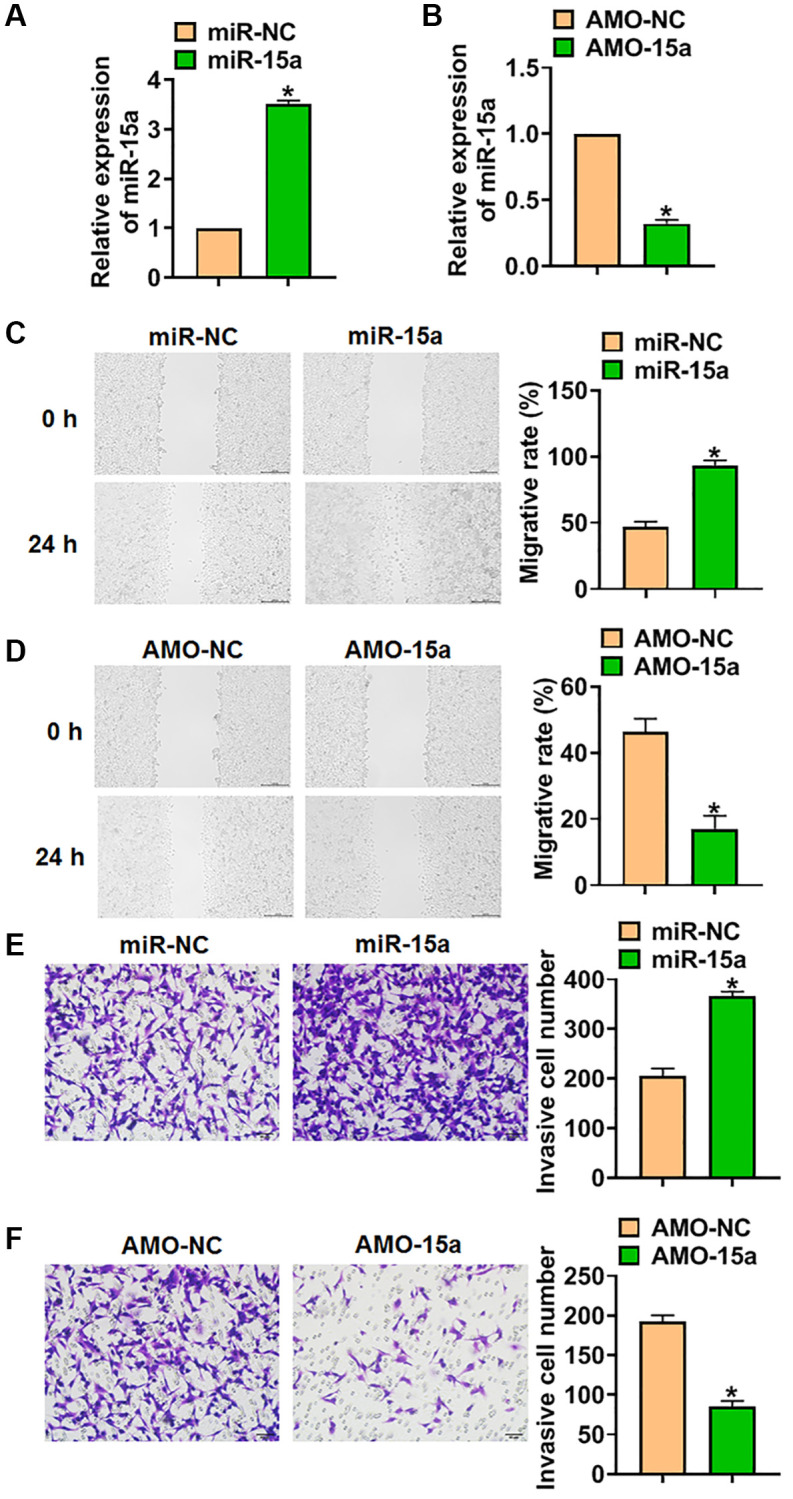
**Silencing of miR-15a inhibited the malignancy of SHG139 cells.** (**A**) SHG139 cells were transfected with miR-15a or its negative control (miR-NC), and qRT-PCR used to test the transfection efficiency. (**B**) AMO-15a or its negative control (AMO-NC) were transfected into SHG139 cells, and miR-15a level in SHG139 cells were detected. (**C**) Wound healing assay was used to detect cell migration. Scale bar, 200 μm. (**D**–**F**) Transwell assay was performed to identify the invasion of SHG139 cells. Scale bar, 50 μm. Data were expressed as mean ± SD.^*^P < 0.05.

To clarify the role of miR-15a in glioma progression, SHG139 cells were transfected with miR-15a mimics or AMO-15a to force or silence miR-15a expression. And qRT-PCR analysis showed that miR-15a transfection increased the level of miR-15a (Figure 2A), while AMO-15a transfection decreased miR-15a expression in SHG139 cells (Figure 2B). Considering the critical role of migration and invasion in tumor development, we tested them using wound healing and transwell assay. The data indicated that miR-15a mimics promoted migration and invasion of SHG139 cells (Figure 2C and 2E), while AMO-15a inhibited migration and invasion of SHG139 cells (Figure 2D and 2F).

### miR-15a inhibited Smad7 expression

**Figure 3:**
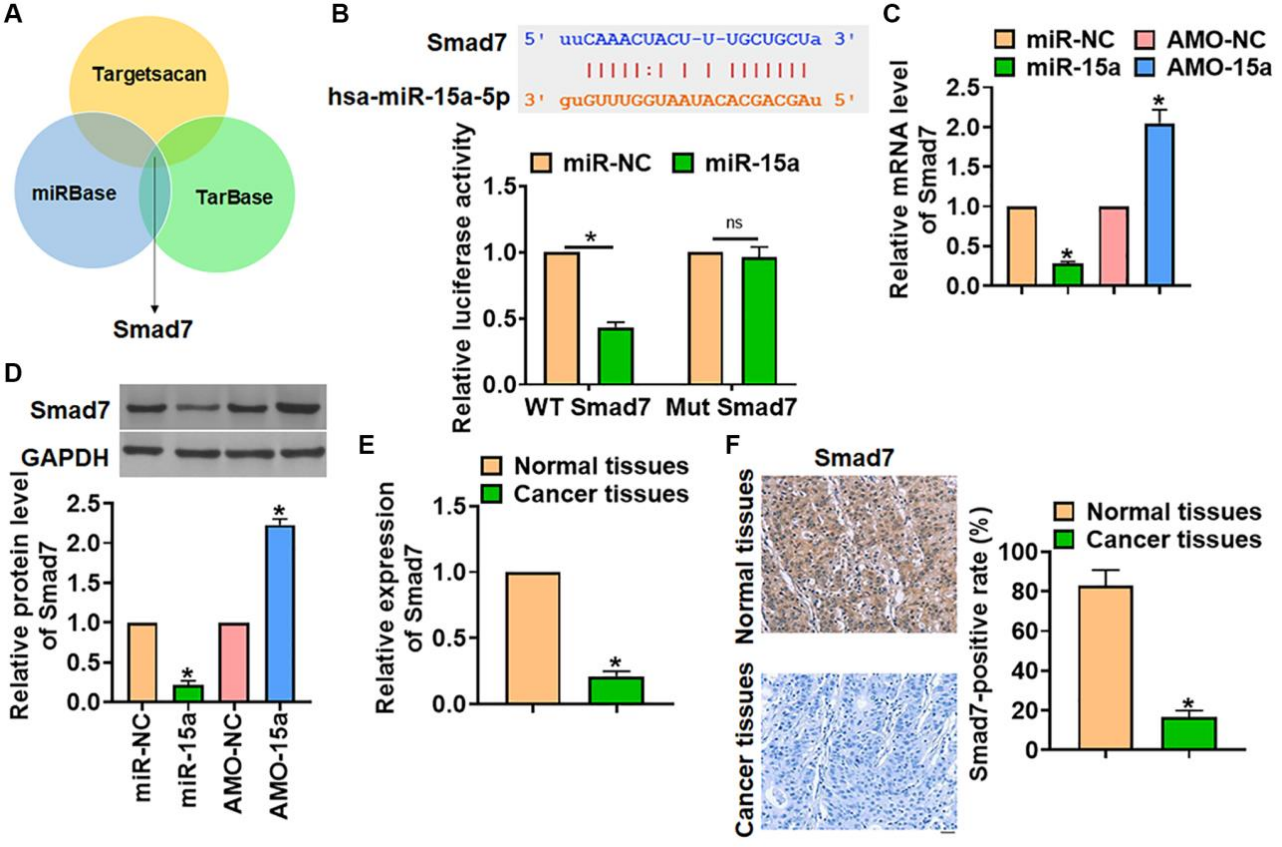
**Smad7 was the target of miR-15a in glioma progression.** (**A**) The miRBase, Targetscan and Tarbase were used to search the predicted target of miR-15a, and Smad7 has the has the greatest potential. (**B**) The binding bases between miR-15a and Smad7 (upper), and luciferase assay used to test the activity of WT Smad7 and Mut Smad7 (lower) in HEK193 cells. SHG139 cells were transfected with miR-15a or AMO-15a, and the mRNA (**C**) and protein (**D**) level of Smad7 was explored. (**E**) The mRNA expression of Smad7 in in adjacent normal tissues and cancer tissues of glioma was determined. (**F**) IHC staining used to examine the expression of Smad7 in different grades of glioma tissues, and relative Smad7 positive area was calculated. Scale bar, 30 μm. Data were expressed as mean ± SD.^*^P < 0.05.

To illustrate the mechanism of miR-15a modulating glioma, three databases (miRBase, Targetscan and Tarbase) were used to search the predicted target of miR-15a, which showed that Smad7 might be the target of miR-15a (Figure 3A). Then, luciferase assay was used to test the binding between miR-15a and Smad7. The data showed that miR-15a, not miR-NC, inhibited the luciferase activity of the 3′UTR of WT-Smad7 (Figure 3B). Also, miR-15a inhibited the mRNA and protein level of Smad7, while AMO-15a increased Smad7 expression (Figure 3C and 3D). Then, we analyzed the expression of Smad7 in glioma tissues. The qRT-PCR and IHC data indicated a prominent increase in cancer tissues of glioma (Figure 3E and 3F).

### Inhibiting of miR-15a restrained the malignancy of glioma cells via increasing Smad7 and EMT pathway

**Figure 4:**
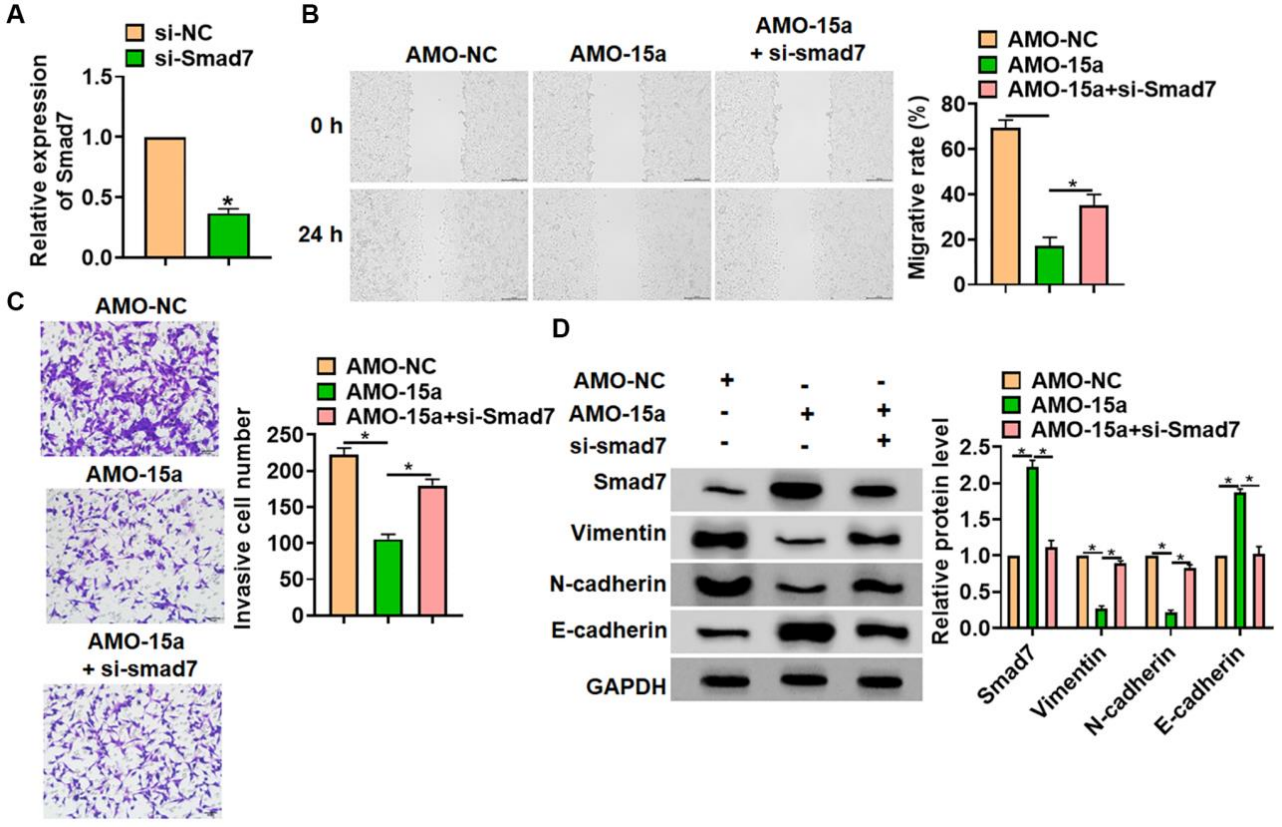
**Inhibiting Smad7 blocked the inhibitory effect of AMO-15a in SHG139 cells.** (**A**) The siRNA of Smad7 (si-Smad7) or its negative control (si-NC) was transfected into SHG139 cells, and the reduction efficiency was examined. SHG139 cells were co-transfected with si-Smad7 and AMO-15a. (**B**) Wound healing assay was used to detect cell migration. Scale bar, 200 μm. (**C**) Transwell assay was performed to identify the invasion of SHG139 cells. Scale bar, 50 μm. (**D**) Western blot was used to tested the Smad7 protein expression and EMT relative markers (Vimentin, N-cadherin and E-cadherin). Data were expressed as mean ± SD.^*^P < 0.05.

Then, we explored whether miR-15a suppressed glioma via modulating Smad7. The small interfering RNA of Smad7 (si-Smad7) was constructed, the si-Smad7 transfection reduced the Smad7 expression in SHG139 cells (Figure 4A). The SHG139 cells were co-transfected with AMO-15a and si-Smad7. Following function experiments showed that Smad7 abolished the inhibiting effect of AMO-15a on cell migration and invasion (Figure 4B and 4C). As well, we examined the epithelial-mesenchymal transition (EMT) markers expression (Vimentin, N-cadherin and E-cadherin). The western blot results indicated that AMO-15a reduced the expression of Vimentin and N-cadherin, but induced E-cadherin (Figure 4D). However, si-Smad7 remitted the effects of AMO-15a on EMT markers level (Figure 4D). These data revealed that deletion of miR-15a restrained the malignancy of glioma cells and activated EMT pathway via modulating Smad7.

### miR-15a inhibitor inhibited glioma growth *in vivo*

**Figure 5:**
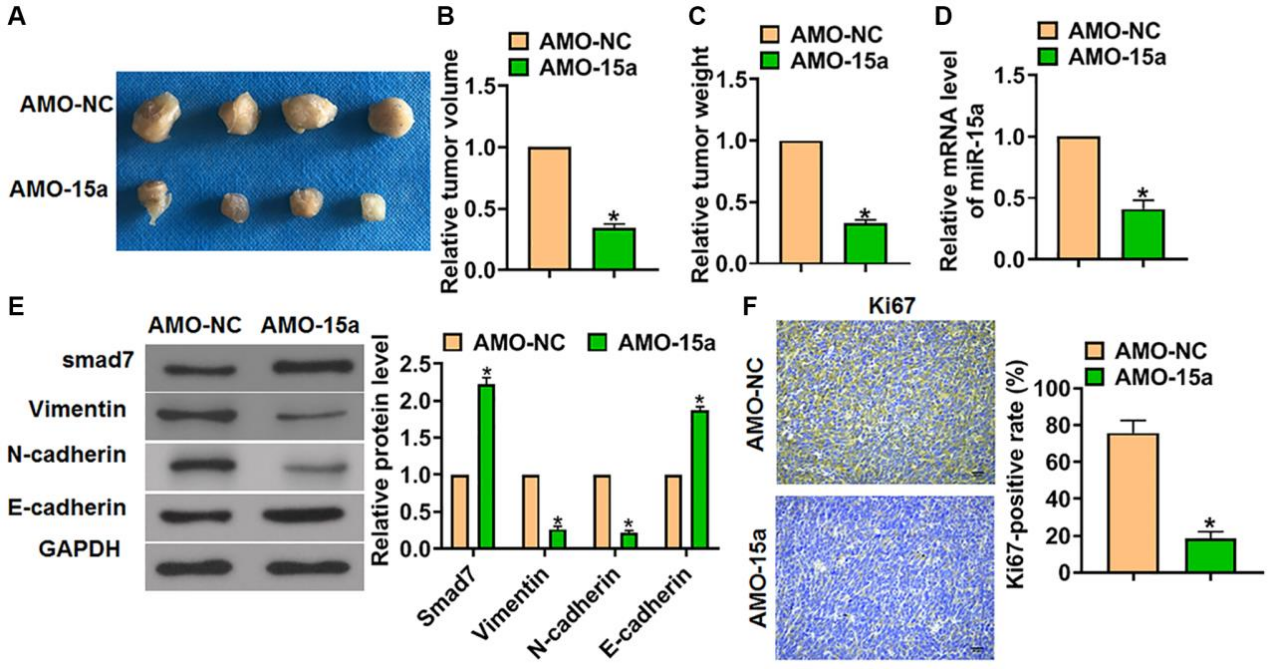
**Suppressed the expression of miR-15a prevented tumorigenesis *in vivo*.** The SHG139 cell lines stably expressing AMO-15a were inoculated into nude mice to establish glioma mouse model. (**A**) Representative images of separated tumors from nude mice after 30 days of inoculation. The relative tumor volume (**B**) and weight (**C**) was calculated. (**D**) The expression of miR-15a in tumors was tested. (**E**) The protein expression of Smad7 and EMT relative markers (Vimentin, N-cadherin and E-cadherin) was tested by western blot. (**F**) The Ki67 expression was test by IHC. Scale bar, 50 μm. Data were expressed as mean ± SD.^*^P < 0.05.

Finally, the function of miR-15a in glioma progression was proved *in vivo*. We constructed SHG139 cell lines stably expressing AMO-15a, and the cells were inoculated into nude mice to establish glioma mouse model. The tumors were separated after 30 days of inoculation (Figure 5A), and we measured tumor volume and weight. The data showed that AMO-15a decreased the tumor volume and weight (Figure 5B and 5C). The expression of miR-15a in tumors was tested by qRT-PCR (Figure 5D). Then, the western blot results showed that AMO-15a promoted Smad7 protein level in tumors (Figure 5E). As well, AMO-15a subdued the activation of EMT pathway, which was exhibited by the downregulation of Vimentin and N-cadherin, and the up-regulation of E-cadherin (Figure 5E). Moreover, Ki67, the proliferation marker, was determined by IHC, which showed that AMO-15a inhibited the Ki67 positive area (Figure 5F).

## DISCUSSION

Metastasis of malignant tumors is an important reason threatening the survival of patients. In the process of tumor metastasis, tumor cells flee from the primary tumor, and metastasize to other tissues and organs in the body along with blood or lymph [19]. Moreover, the occurrence of EMT in tumor cells will improve the migration and invasion ability of tumor cells, and further reduce the survival rate of patients [20]. A large number of studies have shown that miRNAs are involved in the process of tumor migration and invasion. In the present study, we found that miR-15a was up-regulated in glioma tissues, and deletion of miR-15a inhibited the malignancy of glioma cells and EMT pathway via targeting Smad7 both *in vitro* and *in vivo*.

MiRNA is a molecule that regulates the post-transcriptional level of gene expression and protein translation, and plays a pivotal role in the progression of tumors. More and more studies have shown that abnormally expressed miRNAs can be involved in the occurrence and development of tumors as proto- oncogenes or tumor suppressor genes [21]. It was found that the abnormal expression of miRNAs can significantly affect the progression of glioma. For example, miR-1908 is expressed significantly higher in glioma tissues and is associated with poor prognosis in glioma patients [22]. At the same time, miR-1908 targeted PTEN levels and was involved in glioma development as an oncogene. In the present study, we found that miR-15a was significantly increased in glioma tissues. The high level of miR-15a indicated a poor prognosis of glioma patients. MiR-15a located on human chromosome 13q14.2 (https://www.ncbi.nlm.nih.gov/gene/406948), and Malzkorn et al. also found an increase of miR-15a in glioma tissues [23].

Although there have been many studies on miR-15a [24], the specific mechanism of miR-15a in glioma has not been reported. In order to further clarify the mechanism of miR-15a in glioma, we verified the functional changes of glioma cells at the cellular level. The data indicated that overexpression of miR-15a promoted the migration and invasion of glioma cells, while deletion of miR-15a inhibited the malignancy of glioma cells. These results suggested that miR-15a promoted the migration and invasion of glioma cells, which also proved the crucial role of miR-15a in glioma development. Our further studies showed that Smad7 might be a potential target of miR-15a in glioma progression. Smad7 is the downstream gene of the TGF-β pathway, which antagonizes TGF-β receptor I and prevents the phosphorylation of Smad2/3, thus blocking the signal transduction process [25, 26]. Smad7, as a tumor suppressor gene, is low expressed in various cancers, including colorectal cancer [27]. Present data indicated that Smad7 was a direct target of miR-15a, and Smad7 was down-regulated in glioma tissues and cells. Besides, miR-15a inhibitor inhibited the EMT pathway and the migration and invasion of glioma cells. The silencing of Smad7 reversed the effects of miR-15a inhibitor in EMT pathway and glioma progression, which revealed that miR-15a modulated glioma progression via targeting Smad7. Finally, we performed animal experiments to verify the role of miR-15a *in vivo*. Consistent with the results of the cell experiments, the miR-15a inhibitor suppressed glioma growth *in vivo*.

Nevertheless, there were some limitations of present study. Present study only showed a potential molecular mechanism in glioma development. MiR-15a-Smad7 axis cannot be proved as the only pathway in glioma development, and Smad7 might only be an intermediary of miR-15a in modulating EMT pathway. In subsequent studies, we will further explore the role of miR-15a and further explore the evidence of its regulation of EMT signaling.

In conclusion, deletion of miR-15a inhibited the activation of EMT signaling via targeting Smad7, thus suppressed the tumorigenesis and tumor growth of glioma. The present study might provide new ideas and new targets for tumor immunotherapy of glioma, and showed the theoretical value and potential application prospect.

## MATERIALS AND METHODS

### Clinical specimens

A total of 100 cases of glioma diagnosed and treated in Cangzhou Central Hospital from 2013 to 2018 were selected. All patients did not receive radiotherapy, chemotherapy, and immunization before liver tissue collection, and all tissues were confirmed by pathological examination. The studies involving human participants were reviewed and approved by our hospital. The patients/participants provided their written informed consent to participate in this study.

### Cell culture and transfection

The normal astrocytes and glioma cell lines (U87 and SHG139) were purchased from American Type Culture Collection. The cells were cultured with DMEM (Hyclone) and 10% FBS medium, and maintained under a humidified atmosphere containing 5% CO_2_ at 37°C. To confirm the role of miR-15a on glioma progression, 20 nM miR-15a mimics/anti-miRNA oligonucleotide of miR-15a (AMO-15a) its negative control (miR-NC/AMO-NC) was transfected into SHG139 cells using Lipo2000. To confirm the role of Smad7 on miR-15a modulated glioma, 2000 ng Small interfering RNA of Smad7 (si-Smad7) or its negative control (si-NC) was co-transfected into SHG139 cells with AMO-15a. The miR-15a mimics, AMO-15a and si-Smad7 were constructed and purchased from GenePharma Co., Ltd (Shanghai, China).

### Western blot

The tissues or cells in each group were collected and treated with RIPA lysate. Then, the protein concentration was evaluated by BCA kit (Beyotime, China). And the proteins in the loading buffer were separated by SDS-PAGE and transferred to the PVDF membrane. The membrane carrying proteins was sealed with blocking fluid and was incubated with Smad7 (1: 1000), Vimentin (1: 500), N-Cadherin (1: 500), E-cadherin (1: 500) and GAPDH (1: 1000) overnight at 4°C. The next day, HRP-labeled secondary antibody (1: 5000) was added and incubated at room temperature for 2 h. The membrane was photographed by ECL exposure and analyzed the gray value of the target bands. All primary antibodies were purchased from Wuhan Sanying (China).

### Quantitative real-time PCR (qRT-PCR)

The RNA Isolation Kit was used to extract total RNA from tissues and cells, and the concentration and quality of the purified RNA were measured using Nanodrop 2000/2000C spectrophotometer. RNA was reverse transcribed into cDNA template at the following reaction temperatures: 37°C 15 min, 50°C 5 min, 98°C 5 min, 4°C hold. The cDNA was stored at −20°C. Then the primers synthesized by Shanghai Jikegein Company were used for quantitative PCR. The reaction system of 20 μL was prepared. The reaction was carried out according to the following steps: pre-denaturation at 95°C for 15 min, denaturation at 95°C for 15 s, and annealing extension at 60°C for 1 min for 35–40 cycles. All mRNAs were standardized by GAPDH, and analyzed by 2^−ΔΔCT^.

### Wound healing assay

The logarithmic growth cells were inoculated in the 6-well plate, and the next day, the 6-well plate was removed. When the cell density of the adherent wall reached more than 90% of the fusion area, 10 μL sterile gun was used at the bottom of each hole, and three parallel lines were drawn along the diameter of each hole to ensure the uniform thickness of the parallel line in each hole, and the angle and strength of the lines should be consistent. Gently rinsed with PBS twice to remove the dead cells floating after scratch. At the same time, to remove the influence of cell proliferation, the experiment was replaced with 1% FBS DMEM culture medium and placed in a cell culture incubator at 37°C and 5% CO_2_. Experiments were conducted in 6 groups, with 3 compound holes in each group. Under an inverted microscope, the width of the scratch and the speed of scratch healing were observed, and the uniform cell density was marked. Photos were taken at 0 h and 24 h, respectively, and the scratch width was measured at the same time.

### Transwell assay

Transwell chamber with matrix glue is placed in 24-well plate in order to make matrix glue. Cells were resuspended in serum-free DMEM medium, and the count was adjusted to 3 × 10^5^/mL.200 L cell suspension was added to the small chamber, and the cells were incubated at 37°C for 20 min. The Transwell chamber was taken out, the culture medium was discarded, 200 μL of cell suspension was added, and 10% FBS DMEM culture medium 500 μL was added in the next chamber and placed in a cell incubator at 37°C and 5% CO_2_. Each set has 3 multiple holes. After 36 h, each chamber was taken out and washed twice with PBS, and the matrix glue and uninvaded cells at the bottom of the upper chamber were wiped off with a dry cotton swab. After methanol was added to the lower chamber and fixed for 30 min, the cells at the bottom of the small outdoor room were completely immersed in methanol, and then stained with 0.1% crystal violet in the dark for 30–60 min. Then, the cells were gently rinsed with PBS twice, and the filter paper was sucked dry or naturally dried. Under an inverted microscope, 5 randomly selected fields were observed, photographed and counted.

### Immunohistochemical (IHC) staining

Liver tissues from patients or lymph node tissues from mice were collected, fixed with paraformaldehyde, embedded in paraffin and sectioned, dewaxed to distilled water, and hydrated with gradient ethanol. The slides were immersed in 0.01M sodium citrate solution and heated until boiling. Soak with 3% H_2_O_2_ for 10 min; 10% goat serum was sealed for 10 min, and primary antibodies Ki 67 (1:200) purchased from Wuhan Sanying (China) were incubated at 4°C overnight. The second antibody was incubated at 37°C for 30 min the next day. Drop 100 μl DAB onto the tissue. After staining with hematoxylin for 5 min, differentiation with hydrochloric acid and alcohol, washing with water, soaking with ammonia in return blue for 3 min. Then, the gradient alcohol was used for dehydration, the neutral resin was added to seal the slice. Microscopic pictures were taken to calculate the number of positive cells.

### Luciferase assay

The reporter plasmids containing the 3′UTR of wild type (WT) or mutant (Mut) Smad7 were synthesized and co-transfected into HEK293 cells (1 × 10^5^/well) with miR-15a/miR-NC using lipo 2000. At 48 h after transfection, fluorescence intensity was measured according to the Dual-Luciferase^®^Reporter Assay System (Promega) specification.

### *In vivo* tumorigenesis experiment

The SHG139 cells stably expressing AMO-15a were constructed, and SHG139 cells (1 × 10^6^) were adjusted in 0.1 ml normal saline and then divided into different packs. 40 male BALB/C mice aged 6–8 weeks were selected and subcutaneously injected with mixed cells on the right lower limb to establish a mouse liver cancer model. After 4 weeks, the tumor was removed and a follow-up experiment. Tumor volume (mm^3^) = (length × width^2^)/2. The animal study was reviewed and approved by our hospital.

### Statistical analysis

GraphPad Prism software was used for statistical analysis. Data were expressed as mean ± SD. Analysis of variance (ANOVA) was used to compare the data between groups, and then Bonferroni modified *t*-test or Dunnett test was used to evaluate whether there was any difference between groups. *P* < 0.05 was considered significant difference.
